# Australian Notes

**Published:** 1920-11-06

**Authors:** 


### AUSTRALIAN NOTES.

### Value of Australian Indigenous Animals.

Dr. Colin MacKenzie, of Collins Street, Melbourne, whose remarks upon the value of Australia's native animals were quoted in these columns some time ago, has returned to the charge in a column article in the *Argus* wherein he pleads for benefactions not only to prevent their extinction but to encourage their increase, otherwise he fears that in so short a time as another twenty years they will become extinct. Their value, Dr. MacKenzie states, lies a good deal in their human resemblances, and also their differences, one of these being that no instance of cancer in an Australian animal has ever been discovered. In addition, Dr. MacKenzie states that of the numerous ductless glands whose uses are still unknown possessed in common by humanity and the Australian marsupials and monotremes, the "vermin," as the law classes the Australians, have them better developed than any other known animals.

### Help to Scientific Research.

Dr. Mackenzie is now on his way to England, taking with him a large number of anatomical specimens, those illustrating the three ductless glands, new to science, discovered in the platypus, and a good deal of matter bearing on the lymphoid tissues and the curious development of the thyroid gland in the kangaroo. Dr. Mackenzie's object in visiting the Mother Country is to visit the Hunterian Museum of the Royal College of Surgeons (of which he is a member), and to confer with authorities there as to the comparative anatomy of the Australian fauna and its application to medical research. For the past two years Dr. Mackenzie has been working on this matter at the "Australian Institute of Anatomical Research," which he founded. He has enlisted the sympathies of the State Government. Dr. Mackenzie, on behalf of the above-mentioned Institute, has been granted, by the Lands Department, the use of an area of 75 acres of land in the Healesville district, at a peppercorn rental, on condition that the land is used for scientific research only, and occupied only by persons engaged in such research, and by the caretaker. This small territory will make it possible to supplement and extend the work already done, and, as the work develops, a larger area may be granted.

### A Jubilee Appeal Fund.

A short time ago the Alfred Hospital, Melbourne, entered upon its Jubilee year. The foundation-stone of the institution was laid on March 6, 1869, by H.R.H. Prince Alfred, Duke of Edinburgh, hence its name. It was opened for in-patients in May 1871, since when 75,000 have been treated within its wards, while more than six times that number have received outdoor treatment. The "Alfred" was the first general hospital in Victoria to open its doors to victims of the Red Plague, some thousands of whom have been cured or relieved. In view of the necessity that has arisen to increase the accommodation for both patients and nurses, the management is launching an appeal for £51,000, the estimated amount that will be needed. The President of the hospital (Senator Fairbairn) has led the response with a cheque for £1,000. Mr. R. Chambers Norman, for thirty-one years secretary and superintendent of the hospital, is the honorary organiser of the Appeal Fund.

